# Glutathione peroxidase 1 deficiency attenuates concanavalin A-induced hepatic injury by modulation of T-cell activation

**DOI:** 10.1038/cddis.2016.95

**Published:** 2016-04-28

**Authors:** D H Lee, D J Son, M H Park, D Y Yoon, S B Han, J T Hong

**Affiliations:** 1 Medical Research Center, College of Pharmacy, Chungbuk National University, Osongsaengmyeong 1-ro 194-31, Osong-eup, Heungduk-gu, Cheongju, Chungbuk 361-951, Republic of Korea; 2 Department of Bioscience and Biotechnology, Bio/Molecular Informatics Center, Konkuk University, 1, Hwayang-dong, Gwangjin-gu, Seoul 143-701, Republic of Korea

## Abstract

Concanavalin A (Con A)-induced hepatitis model is well-established experimental T cell-mediated liver disease. Reactive oxygen species (ROS) is associated with T-cell activation and proliferation, but continued ROS exposure induces T-cell hyporesponsiveness. Because glutathione peroxidase 1 (Gpx1) is an antioxidant enzyme and is involved in T-cell development, we investigated the role of Gpx1 during Con A-induced liver injury in Gpx1 knockout (KO) mice. Male wild-type (WT) mice and Gpx1 KO mice were intravenously injected with Con A (10 mg/kg), and then killed after 8 h after Con A injection. Serum levels of aspartate transaminase and alanine transaminase were measured to assess hepatic injury. To identify that Gpx1 affects T cell-mediated inflammation, we pretreated Gpx1 inhibitor to Human Jurkat T cells then treated Con A. Con A-induced massive liver damage in WT mice but its damage was attenuated in Gpx1 KO mice. Con A-induced Th1 cytokines such as tumor necrosis factor-*α* (TNF-*α*), interferon-*γ* (IFN-*γ*) and interleukin (IL)-2 were also decreased in the liver and spleen of Gpx1 KO mice compared with WT mice. In Jurkat T cells, Con A-induced mRNA levels of IL-2, IFN-*γ* and TNF-*α* were downregulated by pretreatment of Gpx inhibitor, mercaptosuccinic acid. We also observed that Gpx1 KO mice showed increasing oxidative stress in the liver and spleen compared with WT mice. These results suggest that Gpx1 deficiency attenuates Con A-induced liver injury by induction of T-cell hyporesponsiveness through chronic ROS exposure.

Autoimmune hepatitis (AIH) is an inflammatory disease of the liver by unknown cause that occurs in children and adults of all ages,^1^ characterized by the presence of interface hepatitis and portal plasma cell infiltration on histologic examination, hypergammaglobulinemia, and on loss of self-tolerance leading to appearance of autoantibodies.^2^ However, pathogenic mechanisms of AIH remain obscure. Among the evolved AIH research models such as Concanavalin A (Con A), lipopolysaccharide (LPS) and LPS with d-galactosamine (GalN)-induced models, Con A animal is the most used model and for induction of AIH, because Con A-induced hepatitis model possesses a remarkable change in transaminase level, inflammatory cytokines and immune cells.^3^ Con A-induced hepatitis model is a well-established experimental murine model mimicking human T cell-mediated liver disease.^4^ Con A administration induces severe hepatitis through massive T-cell infiltration, necrosis and apoptosis in the liver of animals.^5^ T lymphocytes activation by intravenous injection of Con A in mice leads to infiltration of CD4+ T lymphocytes in the liver tissue, and infiltrated CD4+ T lymphocytes secrete a large amount of cytokines such as tumor necrosis factor-*α* (TNF-*α*), interferon-*γ* (IFN-*γ*) and interleukin (IL)-2.^6^ The CD4 neutralization protects from Con A-induced liver injury in BALB/c mice.^4^ However, CD8 neutralization shows minor attenuation of liver injury induced by Con A.^3^ These results suggest that CD4+ T helper (Th) cells, but not cytotoxic CD8+ T cells, were major factor in Con A-induced liver injury.

Oxidative stress is closely associated with inflammation. Reactive oxygen species (ROS) are highly reactive and likely to destroy biological structure, promoting cellular damage and progression inflammatory disease.^7^ However, recently published studies showed opposing the traditional concept on ROS, which is the protective role of ROS in immune-mediated inflammatory disease.^8^ Mice with low concentration of ROS due to defects of ROS-producing enzyme such as Ncf1 are more susceptible to autoimmune disease.^9^ Humans with lower levels of ROS such as patients with chronic granulomatous disease (CGD) than normal persons are also more susceptible to autoimmune disease.^10, 11^ Moreover, the pharmacological antioxidant *N*-acetylcystein (NAC) enhances T-cell function and proliferation by inhibition of nuclear factor-*κ*B (NF-*κ*B).^12, 13^ Cermerski *et al.*^14^ reported that T lymphocytes that are isolated form peripheral blood rendered hyporesponsive by exposure to an oxidative environment through inactivation of phospholipase C-*γ* (PLC*γ*). These results suggest that ROS level is important in T-cell activation and proliferation.

Glutathione peroxidase 1 (Gpx1) is first identified as a selenium-dependent enzyme in 1973 and has a role as antioxidant enzyme.^15^ Gpx1 is expressed in most cell types and reduces hydrogen peroxide or lipid peroxides using electrons provided by reduced glutathione (GSH).^16^ Gpx1 may have a limited antioxidant role under normal physiological condition because Gpx1-deficient mice are healthy and fertile.^17^ However, among three important intracellular redox systems such as NADPH/NADP, thioredoxin_red_/thioredoxin_ox_ and GSH/oxidized glutathione (GSSG), cellular GSH levels are 1000-fold higher than other redox couples.^18^ Thus, it is suggested that Gpx1 may critically affect intracellular redox reaction. According to the recent report, Gpx1 is involved in T -cell development. Dworkin *et al.*^19^ reported that blood selenium level and Gpx1 activity were significantly decreased in patient with acquired immunodeficiency syndrome such as AIDS compared with healthy controls. Kim *et al.*^20^ reported that dextran sodium sulfate-induced colitis was attenuated in Gpx1 and catalase double knockout (KO) mice through enhancing regulatory T-cell function. Won *et al.*^21^ also reported that Gpx1 KO mice showed attenuation of allergen-induced airway inflammation through suppressing Th2 and Th17 cell development. These results suggest that Gpx1 is involved in regulation of T-cell development and function.

In the present study, we investigated whether Gpx1 deficiency affects Con A-induced liver injury through modulation of T-cell activation. Therefore, we developed a model of Con A-induced acute liver injury in Gpx1-deficient mice and investigated the role of Gpx1 in T cell-mediated hepatic injury.

## Results

### Gpx1 deficiency protects from Con A-induced liver injury

**Figure 1:**
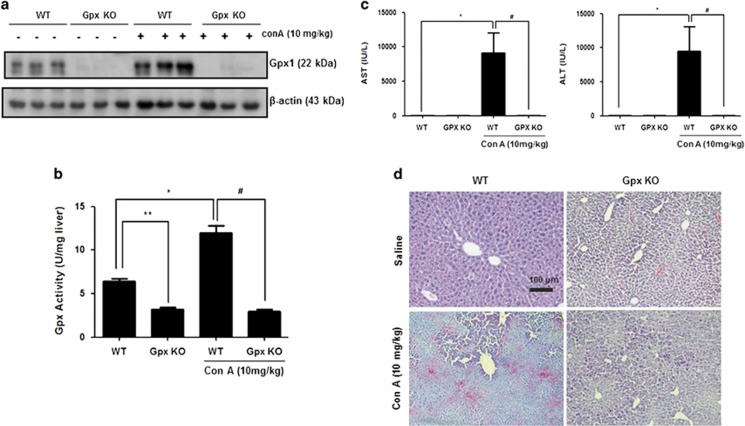
Gpx1 deficiency protects from Con A-induced liver injury. (**a**) The expression of Gpx1 in the liver of WT and Gpx1 KO mice with Con A administration or without. (**b**) The expression of Gpx1 in the liver of WT and Gpx1 KO mice with Con A administration or without. *n*=8 per group; means±S.E.M., *P<0.05, WT mice without Con A administration *versus* WT mice with Con A administration, **P<0.05, WT mice without Con A administration *versus* KO mice without Con A administration, ^#^*P*<0.05, WT mice with Con A administration *versus* Gpx1 KO mice with Con A administration. (**c**) Serum AST and ALT levels in WT and Gpx1 KO mice with Con A administration or without. *n*=8 per group; means±S.E.M., *P<0.05, WT mice without Con A administration *versus* WT mice with Con A administration, ^#^*P*<0.05, WT mice with Con A administration *versus* Gpx1 KO mice with Con A administration. (**d**) Liver sections of WT and Gpx1 KO mice with Con A administration or without were stained with hematoxylin and eosin (H&E) (scale bars, 100 *μ*m)

**Figure 2:**
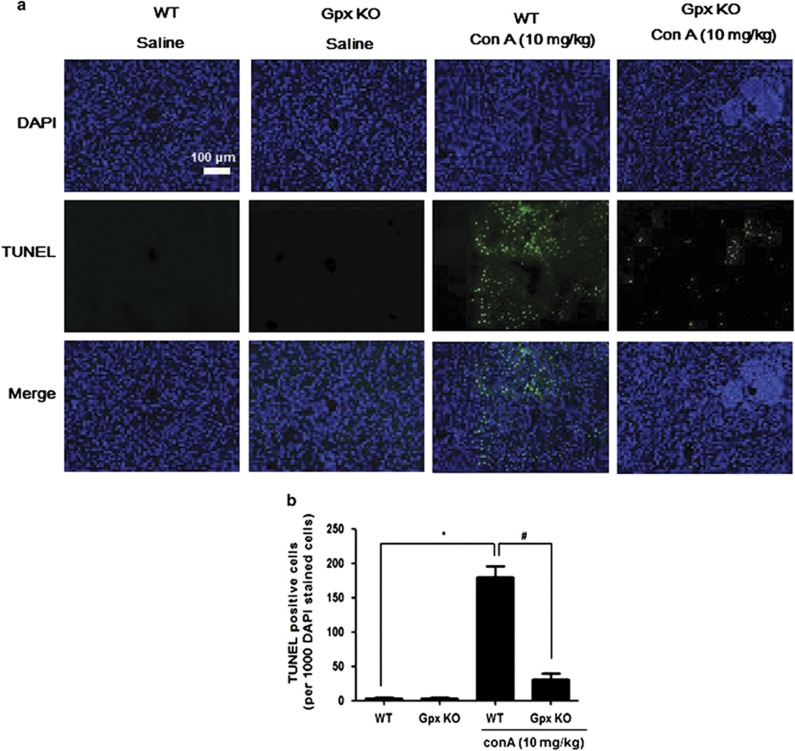
Gpx1 deficiency attenuates Con A-induced hepatic damage. (**a**) Liver sections of WT and Gpx1 KO mice with Con A administration or without were stained with TUNEL (scale bars, 100 *μ*m) of (**b**) Quantification of TUNEL-positive cells (per 10 000 DAPI-stained cells). *n*=8 per group; means±S.E.M., *P<0.05, WT mice without Con A administration *versus* WT mice with Con A administration, ^#^*P*<0.05, WT mice with Con A administration *versus* Gpx1 KO mice with Con A administration

To investigate the role of Gpx1 in experimental T cell-mediated hepatitis, we injected Con A into WT and Gpx1 KO mice. The expression of Gpx1 in the liver of GPx1 KO mice was decreased compared with WT mice with saline and Con A administration, and it was increased by Con A injection in the liver of WT mice (Figure 1a). Gpx activity in the liver of WT mice also was increased by Con A injection, and it was decreased in the liver of both saline- and Con A-injected Gpx1 KO mice (Figure 1b). Serum alanine transaminase (ALT) and aspartate transaminase (AST) levels after Con A injection were significantly lower in Gpx KO mice compared with WT mice (Figure 1c). Histological analysis of liver exhibited that Gpx1 KO mice are less sensitive to Con A-induced hepatic injury (Figure 1d). Liver tissue sections in WT mice showed massive necrosis in the liver after Con A injection, whereas Gpx1 KO mice injected with Con A showed minor damage. TUNEL assay showed a significant injury in the livers of WT mice injected with Con A, which was markedly attenuated in the livers of Gpx1 KO mice injected with Con A (Figure 2).

### Gpx1 deficiency inhibits Con A-induced cytokine signaling by inhibition of cytokines production in the liver of mice

**Figure 3:**
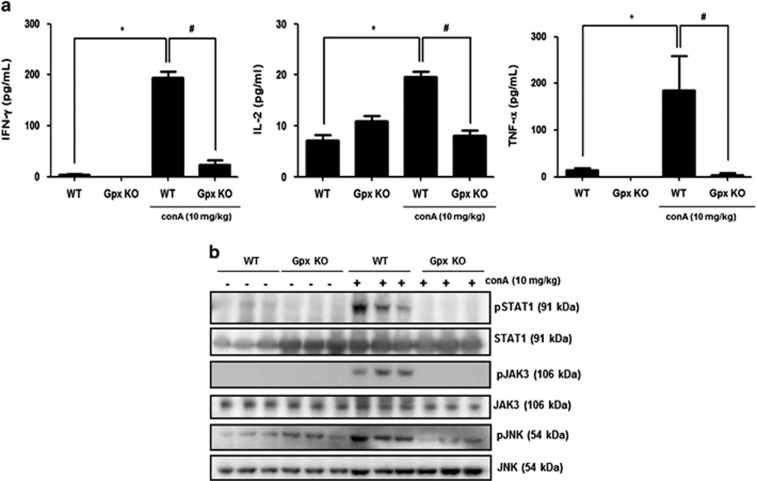
Gpx1 deficiency significantly reduces cytokine production and inhibits its signaling pathway in the liver of mice. (**a**) Cytokine assay of IL-2, IFN-*γ* and TNF-*α* in the liver of WT and Gpx1 KO mice with Con A administration or without. *n*=8 per group; means±S.E.M., *P<0.05, WT mice without Con A administration *versus* WT mice with Con A administration, ^#^*P*<0.05, WT mice with Con A administration *versus* Gpx1 KO mice with Con A administration. (**b**) Immunoblots of STAT1, JAK3 and JNK phosphorylation in the liver of WT and Gpx1 KO mice with Con A administration or without

Th1 cytokines such as IFN-*γ*, IL-2 and TNF-*α* are involved in the Con A-induced liver injury.^3^ We next examined whether Gpx deficiency influences expression level of these cytokines in the liver. As shown in Figure 3a, some major cytokines such as IFN-*γ*, IL-2 and TNF-*α* were increased in the liver of WT mice injected with Con A, whereas those were not increased in the liver of Gpx1 KO mice injected with Con A. Con A-induced liver injury was mediated by phosphorylation of STAT1 induced by IFN-*γ*, which was secreted from Con A-induced CD4 T cells.^5^ STAT1 phosphorylation was increased by Con A administration in the liver of WT mice, whereas its phosphorylation did not increase in the liver of Con A-injected Gpx1 KO mice (Figure 3b). Con A induced T-cell proliferation and activation by increased IL-2 levels.^3^ In the liver of WT mice, phosphorylation of JAK3 which is activated by IL-2 was increased by Con A administration, whereas its phosphorylation did not increase in the liver of Gpx1 KO mice injected with Con A (Figure 3b). TNF-*α*-mediated liver injury correlates with JNK activation.^22^ In the liver of WT mice, phosphorylation of JNK which is activated by TNF-*α* was increased by Con A administration, whereas its phosphorylation did not increase in the liver of Gpx1 KO mice injected with Con A (Figure 3b).

### Influx of effector cells in the liver is reduced in Gpx1 KO mice

**Figure 4:**
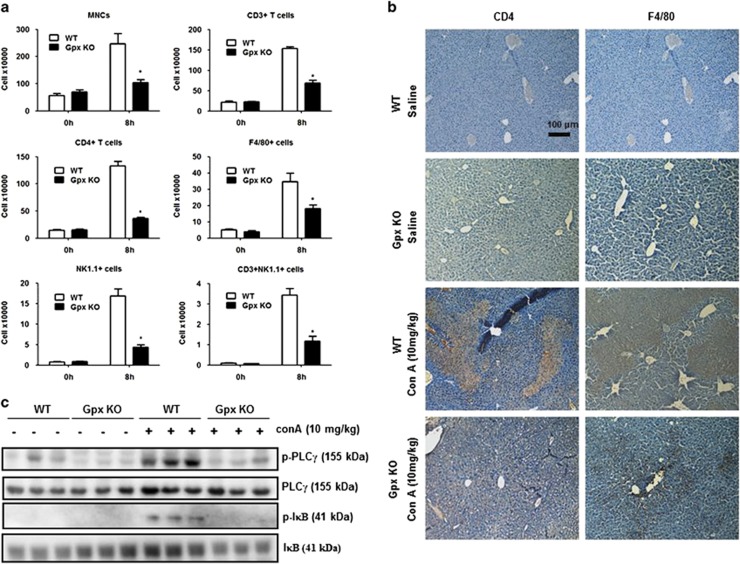
Gpx1 deficiency reduced the influx of CD4+ T cells and macrophages, and inhibits T-cell responses in the liver of mice. (**a**) Flow-cytometry analysis showed that Con A-induced MNCs are markedly decreased in the liver of Gpx1 KO mice. *n*=8 per group; means±S.E.M., *P<0.05, WT mice with Con A administration *versus* Gpx1 KO mice with Con A administration. (**b**) Immunohistochemistry of infiltrated immune cells such as CD4+ T cells and macrophages (F4/80) in the liver of WT and Gpx1 KO mice with Con A administration or without. (**c**) Immunoblots of PLC*γ* and I*κ*B phosphorylation in the liver of WT and Gpx1 KO mice with Con A administration or without

After Con A injection, there was an influx of massive mononuclear cells (MNCs) in the liver.^23^ To investigate whether Gpx1 deficiency was related to Con A-induced influx of MNCs in the liver, we performed flow-cytometry analysis in the liver. As shown in Figure 4a, there was a decrease in the total number of liver infiltrating mononuclear cells, CD3+, CD4+ T cells, F4/80+ macrophages, NK1.1+ NK and NK1.1+CD3+ (NKT-like) cells in the liver of Gpx KO mice in comparison with WT mice. Histological analysis also showed that more CD4+ cells or F4/80+ cells were observed in the livers of Con A-injected WT mice than in those of Con A-injected Gpx1 KO mice (Figure 4b). Con A stimulates IL-2 production in T cells via T-cell receptor and subsequent activation of PLC*γ* and its downstream NF-*κ*B pathway.^24^ PLC*γ* and I*κ*B phosphorylation was increased by Con A administration in the liver of WT mice, whereas those phosphorylation did not increase in the liver of Con A-injected Gpx1 KO mice (Figure 4c).

### Gpx1 modulates Con A-induced CD4+ T cell in the spleen

**Figure 5:**
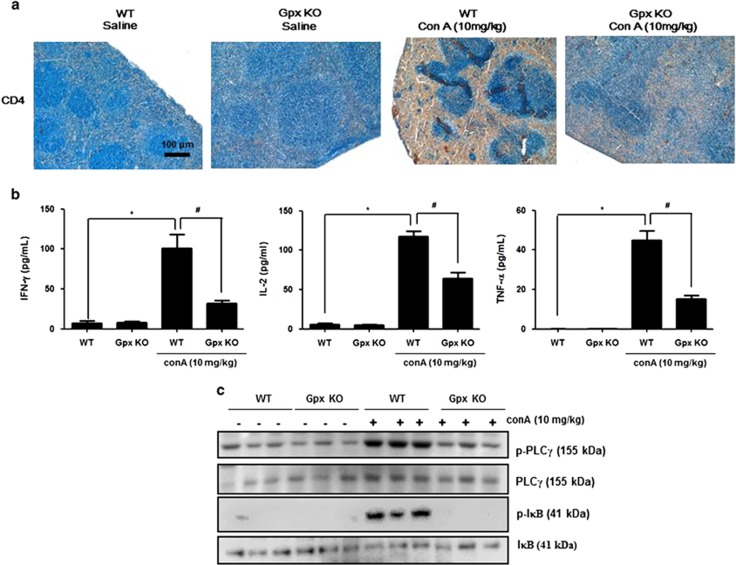
Gpx1 deficiency reduced Con A-induced CD4+ T cells, Th1 cytokines and T-cell responses in the spleen. (**a**) Immunohistochemistry of CD4+ T cells in the spleen of WT and Gpx1 KO mice with Con A administration or without. (**b**) Cytokine assay of IL-2, IFN-*γ* and TNF-*α* in the spleen of WT and Gpx1 KO mice with Con A administration or without. *n*=8 per group; means±S.E.M., *P<0.05, WT mice without Con A administration *versus* WT mice with Con A administration, ^#^*P*<0.05, WT mice with Con A administration *versus* Gpx1 KO mice with Con A administration. (**c**) Immunoblots of PLC*γ* and I*κ*B phosphorylation in the liver of WT and Gpx1 KO mice with Con A administration or without

In Con A-induced hepatic injury, T cells can be classified into two groups such as exogenous and endogenous.^25^ Exogenous T cells originate from the thymus, bone marrow, intestinal tract, spleen and lymph gland, and enter the liver through circulation.^3^ To examine whether Gpx1 deficiency affects activation of exogenous T cell, we analyzed the distribution of CD4+ T cells and levels of Th1 cytokines such as TNF-*α*, IFN*γ* and IL-2 in the spleen. As shown in Figure 5a, CD4-positive cells were more observed in the spleen of Con A-injected WT mice than Con A-injected Gpx1 KO mice. Th1 cytokines such as IFN-*γ*, IL-2 and TNF-*α* were also increased in the spleen of WT mice injected with Con A, whereas those were not increased in the spleen of Gpx1 KO mice injected with Con A (Figure 5b). Moreover, PLC*γ* and I*κ*B phosphorylation was increased by Con A administration in the spleen of WT mice, whereas those phosphorylation did not increase in the spleen of Gpx1 KO mice with Con A administration (Figure 5c).

### Inhibition of Gpx1 induces oxidative stress-induced T-cell hyporesponsiveness and affects Con A-induced T-cell activation and proliferation in human Jurkat T cells

**Figure 6:**
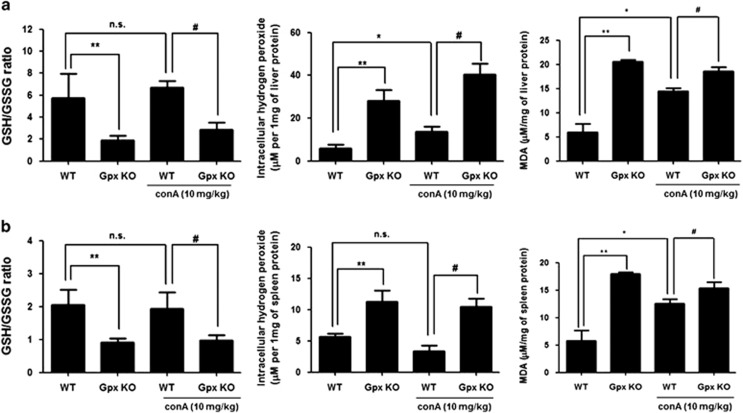
Gpx1 deficiency exhibits low GSH/GSSG ratio, and high level of hydrogen peroxide and lipid peroxidation in the liver and spleen GSH/GSSG ratio, hydrogen peroxide and MDA levels in the (**a**) liver and (**b**) spleen of WT and Gpx1 KO mice with Con A administration or without. *n*=8 per group; means±S.E.M., *P<0.05, WT mice without Con A administration *versus* WT mice with Con A administration, **P<0.05, WT mice without Con A administration *versus* KO mice without Con A administration, ^#^*P*<0.05, WT mice with Con A administration *versus* Gpx1 KO mice with Con A administration

**Figure 7:**
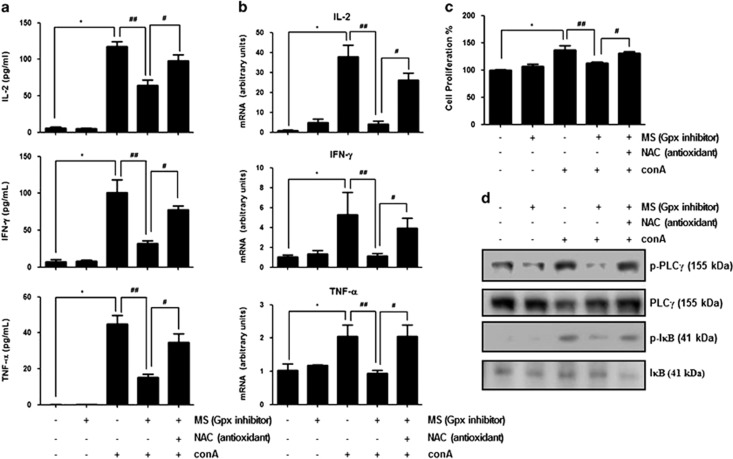
Inhibition of Gpx activity induces T-cell hyporesponsiveness. (**a**) The splenocytes isolated from spleen of WT mice with NAC (5 mM, pharmacological antioxidant) or without, and pretreated MS (0.4 mM) for 48 h, then treated Con A (5 *μ*g/ml) for 8 h. Then, we performed cytokine assay. (**b**) Con A-induced mRNA expressions of Th1 cytokines such as IL-2, IFN-*γ* and TNF-*α* in MS (0.4 mM, Gpx inhibitor)-pretreated Jurkat T cell with NAC (5 mM, pharmacological antioxidant) or without. Values are expressed as the mean±S.E.M. of three different experiments conducted in triplicates. *P<0.05, control *versus* Con A, ^##^*P*<0.05, Con A *versus* pretreatment of MS then Con A, ^#^*P*<0.05, pretreatment of MS then Con A *versus* pretreatment of MS in the presence of NAC then Con A. (**c**) BrdU incorporation assay of Con A-induced cell proliferation in MS (0.4 mM, Gpx inhibitor)-pretreated Jurkat T cell with NAC (5 mM, pharmacological antioxidant) or without using the Cell Signaling BrdU Cell Proliferation Assay Kit. Values are expressed as the mean±S.E.M. of three different experiments conducted in triplicates. *P<0.05, control *versus* Con A, ^##^*P*<0.05, Con A *versus* pretreatment of MS then Con A, ^#^*P*<0.05, pretreatment of MS then Con A *versus* pretreatment of MS in the presence of NAC then Con A. (**d**) Immunoblots of PLC*γ* and I*κ*B phosphorylation in Con A-induced cell proliferation in MS (0.4 mM, Gpx inhibitor)-pretreated Jurkat T cell with NAC (5 mM, pharmacological antioxidant) or without

Continued ROS exposure induces T-cell hyporesponsiveness through irreversible oxidation.^18^ To examine that oxidative stress affects Con A-induced T-cell activation, we evaluated GSH/GSSG ratio and measured level of hydrogen peroxide and malondialdehyde (MDA), which are indicators of oxidative stress.^26^ We observed that Con A injection weakly increased hydrogen peroxide and MDA level but GSH/GSSG ratio did not change by Con A injection in the liver of WT mice (Figure 6a). In the spleen, hydrogen peroxide level and GSH/ GSSG ratio were not changed by Con A injection, but MDA level was weakly increased by Con A in the WT mice (Figure 6b). In contrast to saline-injected WT mice, saline-injected Gpx1 KO mice showed low GSH/GSSG ratio, increased hydrogen peroxide and high MDA level in the liver and spleen (Figure 6). Moreover, Con A-injected mice also showed low GSH/GSSG ratio, increased hydrogen peroxide and high MDA level in the liver and spleen compared with Con A-injected WT mice (Figure 6). These results suggest that Gpx1 deficiency attenuates Con A-induced T-cell activation through continued ROS-induced T-cell hyporesponsiveness. Next, we examined whether inhibition of Gpx1 affects Con A-induced T-cell activation. First, we observed that hydrogen peroxide level was increased by mercaptosuccinylacid (MS) and its increased levels were abrogated by NAC (5 mM) in splenocytes (Supplementary Figure 1a). However, Con A treatment did not affect hydrogen peroxide production in splenocytes (Supplementary Figure 1a). In splenocytes from WT mice, pretreatment of MS (0.4 mM, Gpx inhibitor) for 48 h inhibited Con A-induced cytokines such as IL-2, IFN-*γ* and TNF-*α* (Figure 7a). These inhibitory effects of Con A-induced cytokine production by MS pretreatment were abrogated by NAC (5 mM), a pharmacological antioxidant (Figure 7a). Like splenocytes, hydrogen peroxide level was also increased by MS and its increased levels were abrogated by NAC (5 mM) in Jurkat T cells (Supplementary Figure 1b). However, Con A treatment did not affect hydrogen peroxide production in Jurkat T cells (Supplementary Figure 1b). In Jurkat T cells, the mRNA expression levels of Th1 cytokines such as IL-2, IFN-*γ* and TNF-*α* were increased by Con A treatment (5 *μ*g/ml). However, increased mRNA expression levels of IFN-*γ*, IL-2 and TNF-*α* by Con A were decreased by pretreatment of MS (0.4 mM, Gpx inhibitor) for 48 h in Jurkat cells (Figure 7b). These Con A-induced cytokine production inhibitory effects of MS pretreatment were also abrogated by NAC (5 mM) (Figure 7b). Con A-induced cell proliferation rate was also decreased by pretreatment of MS, and this inhibitory effect was abrogated by NAC (5 mM) in Jurkat cells (Figure 7c). Moreover, Con A-induced PLC*γ* and I*κ*B phosphorylation was decreased by pretreatment of MS (0.4 mM, Gpx inhibitor) for 48 h in Jurkat cells, whereas those phosphorylation did not decrease by NAC (5 mM) in Jrukat cells with pretreatment of MS (0.4 mM, Gpx inhibitor) (Figure 7d). These results suggest that inhibition of Gpx activity inhibits Con A-induced T-cell activation and proliferation through continued ROS-induced T-cell hyporesponsiveness.

## Discussion

ROS is highly associated with immune response, and elevated ROS level promotes cellular damage and progresses inflammatory disease.^7^ However, recently several studies reported a protective role of ROS in immune-mediated inflammatory disease, because an imbalance between ROS generation and scavenging may contribute to abnormal T-cell responses.^18^ Gpx1 is an antioxidant enzyme that is expressed in most cell types and reduces hydrogen peroxide or lipid peroxides using electrons provided by reduced GSH.^16^ Ren *et al.*^27^ reported that selenium-induced increases of activity and expression of Gpx1 promote T-cell responses by Con A in primary porcine splenocytes. We found that Gpx1 expression and activity increased in the liver of mice with Con A administration. Therefore, we speculated that Gpx1 deficiency might ameliorate Con A-induced liver injury. In the present study, we showed that Con A-induced hepatic injury was attenuated in Gpx1 KO mice.

Con A stimulates T-cell response and subsequent T cell-mediated hepatic injury. Con A administration significantly induced hepatic injury in the livers of WT mice, but it was attenuated in Gpx1 KO mice. In the liver tissue of WT mice, massive hepatic necrosis cells were revealed by T cell-mediated inflammation. The level of ALT and AST, a major marker of hepatic damage in the serum, highly elevated in the liver of WT mice with Con A administration but not in the liver of Gpx1 KO mice with Con A administration. These results suggest that Gpx1 deficiency attenuates Con A-induced liver injury.

Some major cytokines involved in Con A-induced liver injury are IL-2, IFN-*γ* and TNF-*α* secreted from CD4+ Th1 cells.^3^ IL-2 induces T-cell proliferation and activation through activation of JAK3. IL-2 KO mice showed immunodeficiency by uncontrolled activation and proliferation of lymphocytes.^28^ IFN-*γ* induces activation of STAT1 and IFN-*γ*/STAT1 have an essential role in CD4 T-cell activation, which directly or indirectly induce liver injury. Hong *et al.*^5^ reported that IFN-*γ* or STAT1 KO mice attenuate Con A-induced hepatic injury. Con A stimulates T cells and increases TNF-*α* production, which binds TNF receptor to induce JNK activation.^22^ JNK activity correlates with Con A-induced hepatocyte damage.^29^ In our results, the levels of IL-2, IFN-*γ* and TNF-*α* were increased by Con A in the liver of WT mice, whereas those levels were not increased in the liver of Gpx1 KO mice with Con A administration. Moreover, activation of STAT1, JAK3 and JNK induced by cytokines such as IL-2, IFN-*γ* and TNF-*α* was inhibited in the liver of Gpx1 KO mice with Con A administration. These results suggest that Gpx1 deficiency attenuates Con A-induced liver injury by inhibition of Th1 cytokines production.

The liver injury in Con A-induced hepatitis is associated with MNCs such as T cells, macrophages and NK cells that infiltrate into liver parenchyma.^4^ In our results, we observed that Gpx1 KO mice exhibited a markedly reduced number of liver-infiltrated MNCs. T cells involved in Con A-induced liver injury are classified as endogenous and exogenous. To examine Con A-induced endogenous T-cell response, we investigated PLC*γ* and I*κ*B phosphorylation in the liver of WT and Gpx1 KO mice. We also investigated immunohistochemistry analysis of CD4+ T cell, PLC*γ* and I*κ*B phosphorylation and evaluation of cytokines level in the spleen, which is one of the originated organ of exogenous T cells. In our results, we observed that Con A-induced CD4+ T cell, Th1 cytokines such as IL-2, IFN-*γ* and TNF-*α*, and PLC*γ* and I*κ*B phosphorylation were decreased in the spleen of Gpx1 KO mice. These results suggest that Gpx1 deficiency inhibits Con A-induced immune cells infiltration into the liver as well as endogenous and exogenous CD4+ T-cell response.

Cermerski *et al.*^14^ reported that oxidative environment induces T-cell hyporesponsiveness through inhibition of PLC*γ* activation induced by T-cell receptor stimulation. In Jurkat T cells, ROS enhances T-cell response through activation of NF-*κ*B;^30^ however, chronic exposure of ROS induces T-cell hyporesponsiveness through NF-*κ*B inactivation.^31^ Interestingly, we observed highly MDA level in the spleen and liver of Gpx1 KO mice compared with WT mice. In our data, Gpx KO mice showed elevated ROS level in the liver but ALT and AST level was not increased in the liver compared with WT mice. In several studies reported that chronic ROS exposure showed not severe liver injury. Chronic ROS exposure by long-term ethanol feeding induces mild steatosis and slightly elevation of serum ALT, with little or no liver inflammation.^32^ Moreover, aging is closely associated with oxidative stress, and aged mice showed reduction of stress tolerance, lower GSH/GSSG ratio and higher MDA levels in the liver compared with young mice; however, serum ALT level in aged mice was not differ from young mice.^33^ In our data, ROS was weakly or not increased by Con A injection in the liver and spleen of WT mice. Moreover, hydrogen peroxide levels in splenocytes and Jurkat T cells did not change by Con A treatment. Shirin *et al.*^34^ reported that ROS may have a secondary role in Con A-induced hepatitis because hepatic oxidative stress was increased 12 h after Con A injection but not earlier. Thus, our results suggest that Gpx1 deficiency makes oxidative environment and continued ROS exposure to T cells in the spleen and liver but affects little or no liver damage. To make T-cell hyporesponsiveness induced by oxidative stress *in vitro* model, Cermerski *et al.*^35^ performed that peripheral blood co-cultured with synovial fluid from rheumatoid arthritis patient which is oxidative environment for 16 h, then eliminated neutrophils. They observed that oxidative stress induces T-cell hyporesponsiveness and this effect recovered by pharmacological antioxidant, NAC or antioxidant enzyme such as catalase.^14^ To examine whether Gpx inhibitor showed a similar effect like Gpx1 deficiency, we treated MS, Gpx inhibitor to splenocytes from spleen of WT mice. Similar to Gpx deficiency, Gpx inhibitor inhibits Con A-induced Th1 cytokines such as IL-2, IFN-*γ* and TNF-*α* in splenocytes from spleen of WT mice. Next, we examined whether Gpx inhibitor affects T-cell response in Jurkat T cells. We observed that mRNA expressions of Th1 cytokines such as IL-2, IFN-*γ* and TNF-*α* as well as cell proliferation rate were inhibited by Gpx inhibitor in Jurkat T cells. These inhibitory effects by Gpx inhibitor were recovered by pharmacological antioxidant, NAC. Con A-induced PLC*γ* activation and its downstream, I*κ*B phosphorylation were also inhibited by Gpx inhibitor, and these inhibitory effects were also recovered by pharmacological antioxidant, NAC. These results suggest that chronic Gpx inhibitor treatment makes oxidative environment and continued ROS exposure-induced T-cell hyporesponsiveness.

In summary, our results suggest that Gpx1 deficiency may prevent Con A-induced liver injury through inhibition of Th1 cytokines production. This effect may result from T-cell hyporesponsiveness induced by continued ROS exposure, which was induced by imbalance between ROS generation and scavenging through inhibition of antioxidant activity of Gpx1.

## Materials and Methods

### Animals

The experimental treatments were carried out according to the guidelines for animal experiments of the Faculty of Disease Animal Model Research Center, Korea Research Institute of Bioscience and Biotechnology (Daejeon, Korea), as well as the Guidelines for the welfare and use of animals in cancer research.^36^ The C57BL/6 wild-type (WT) and Gpx1 KO on the C57BL/6 background mice^17^ were kindly obtained from Sei-Kwan Oh, Ehwa Womens University. Male, age-matched (8 months) Gpx1 KO mice and WT mice were randomly divided into four groups (*n*=8 per group), and were given a single intravenous injection of Con A (Sigma-Aldrich, St. Louis, MO, USA) at 10 mg/kg body weight dissolved in 200 *μ*l of saline. All studies were approved by and performed according to the ethical guidelines by the Chungbuk National University Animal Care Committee (CBNU-523-13-01).

### Serum biochemistries

Mice were anesthetized with an overdose of pentobarbital (100 mg/kg), and blood was taken by heart puncture. Serum levels of AST and ALT were measured at Laboratory Animal Research Center in Chungbuk National University.

### Histological techniques and immunohistochemistry

For histological processing, liver and spleen tissues were fixed in phosphate buffer containing 10% formaldehyde and decalcified with EDTA. Fixed tissues were processed by routine methods to paraffin blocks. Specimens were sectioned at 4 *μ*m and stained with H&E. All specimens in the liver and spleen of mice were fixed in formalin and embedded in paraffin for examination. Sections (4-mm thickness) were analyzed by immunohistochemistry using primary mouse monoclonal antibodies directed against CD4 (1 : 100 dilution), primary rat monoclonal antibody directed against F4/80 (1 : 100), and secondary biotinylated anti-mouse and anti-rat antibodies.

### Flow-cytometry analysis

To isolate hepatic MNCs, the inferior vena cava was cut above the diaphragm and the liver was flushed with cold PBS until it became pale. The connective tissue and the gall bladder were removed, and the liver was minced into small pieces and forced gently through a 200-mm-gauge stainless steel mesh using a sterile syringe plunger and then suspended in 50 ml Roswell Park Memorial Institute (RPMI)-1640 medium containing GlutaMAX-1, 25 mM HEPES and 10% FCS (pH 7.4). The cell suspension was centrifuged at 507 r.p.m. (60 *g*) with the off-brake setting for 1 min at room temperature. The obtained supernatant was transferred to a new tube and centrifuged at 1433 r.p.m. (480 *g*) with the high-brake setting for 8 min at room temperature. The pellet was resuspended in 10 ml 37.5% Percoll in HBSS containing 100 U/ml heparin and then centrifuged at 1907 r.p.m. (850 *g*) with the off-brake setting for 30 min at room temperature. The pellet was resuspended in 2 ml ammonium chloride/Tris-chloride (pH 7.2) (erythrocyte lysing buffer), incubated at room temperature for 5 min, then supplemented with 1 ml FCS and centrifuged subsequently at 1433 r.p.m. (480 *g*) with the high-brake setting for 8 min. Finally, the pellet was resuspended in 1 ml PBS containing 0.1% NaN_3_ (FACS buffer) and prepared for flow cytometry. Hepatic MNCs of WT and Gpx KO mice were screened for various cell surface and intracellular markers with flow cytometry before and at 8 h after Con A injection. Briefly, 1 × 10^6^ MNC was incubated with anti-mouse CD4, anti-mouse CD3, anti-mouse NK1.1 and anti-mouse F4/80, conjugated with fluorescein isothiocyanate (FITC, BD Bioscience, San Jose, CA, USA) or phycoerythrin (PE, BD Bioscience). The stained cells were counted using a BD FACSCalibur, and the results were analyzed with the WinMDI software.

### Measurement of cytokines in the liver and spleen

Liver and spleen tissues homogenized with protein extraction solution (PRO-PREP, iNtRON Biotechnology, Seoul, Korea) and measured the quantity of IL-2, IFN-*γ* and TNF-*α* in total proteins (1 mg) using mouse cytokine assay kit (R&D Systems, Minneapolis, MN, USA).

### Western blot analysis

Homogenized liver and spleen tissues, and splenocytes from spleen of WT mice and Jurkat T cells lysed by protein extraction solution (PRO-PREP, iNtRON Biotechnology) containing protease inhibitor cocktail (Calbiochem, Darmstadt, Germany) and phosphatase inhibitor cocktail (Roche, Basel, Switzerland). Total proteins (30 *μ*g) were separated by SDS-PAGE and transferred onto a PVDF membrane (Millipore, Billerica, MA, USA). The membrane was blocked with 5% skim milk overnight and then incubated with primary antibodies (diluted 1 : 1000) for 1 h at room temperature. The membranes were immunoblotted with following primary antibodies: Phospho-STAT1, Phospho-JAK3, Phospho-JNK, Phospho-PLC*γ*, Phospho-I*κ*B (Cell Signaling Technology, Beverly, MA, USA), STAT1, JAK3, JNK, PLC*γ*, I*κ*B (Santa Cruz Biotechnology, Dallas, TX, USA). After washing with Tris-buffered saline containing 0.05% Tween-20 (TBST), the membrane was incubated with horseradish peroxidase-conjugated secondary antibodies (diluted 1 : 3000) for 1 h at room temperature. Binding of antibodies to the PVDF membrane was detected with enhanced chemiluminescence solution (Amersham Bioscience, Buckinghamshire, UK) and X-ray film (AGFA, Mortsel, Belgium).

### Oxidative stress assay

Hydrogen peroxides were measured according to the manufacturer's instructions (Cell Biolabs, San Diego, CA, USA). GSH, GSSG and MDA were measured according to the manufacturer's instructions (Cayman Chemical, Ann Arbor, MI, USA). To perform assay, the liver tissue and human hepatic cells were homogenized, then normalized to protein concentration.

### Cell culture and regents

The Jurkat human T cells were obtained from the American Type Culture Collection (Manassas, VA, USA). Jurkat T cells were grown at 37 °C in 5% CO_2_-humidified air in RPMI medium that contained 10% fetal bovine serum (FBS), 100 U/ml penicillin and 100 mg/ml streptomycin. RPMI, penicillin, streptomycin and FBS were purchased from Gibco Life Technologies (Grand Island, NY, USA). To investigate whether inhibition of Gpx induces T-cell hyporesponsiveness, we pretreated 0.4 mM MS (Gpx inhibitor, Sigma-Aldrich) for 48 h, then treated Con A (5 *μ*g/ml) for 8 h in Jurkat T cells. To examine whether NAC (pharmacological antioxidant, Sigma-Aldrich) recovered MS-induced T-cell hyporesponsiveness, we pretreated MS (0.4 mM) for 48 h in the absence or presence of 5 mM NAC, then treated Con A (5 *μ*g/ml) for 8 h in Jurkat T cells.

### RNA isolation and quantitative real-time RT-PCR

Total RNA was isolated from Jurkat T cell using Trizol (Invitrogen, Carlsbad, CA, USA). Samples were reverse-transcribed using ProSTAR (Stratagene, La Jolla, CA, USA). Gene expression analysis was performed by RT-PCR using the Quanti Nova SYBR green PCR kit (Qiagen, Valencia, CA, USA).

### Bromodeoxyuridine incorporation assay

We evaluated cell proliferation rate using bromodeoxyuridine (BrdU) cell proliferation assay kit (Cell Signaling, Danvers, MA, USA) according to the manufacturer's protocol.

### Statistical analysis

The experiments were conducted either in triplicate, and all experiments were repeated at least three times with similar results. The data were expressed as the means±S.E.M. Statistical analysis was done using the Student's *t* -test, with the following significance levels: *P<0.05, ^#^*P*<0.05.

## Abbreviations

Con A: Concanavalin A
ROS: reactive oxygen species
Gpx1: glutathione peroxidase 1
WT mice: wild-type mice
Gpx1 KO mice: glutathione peroxidase 1 knockout mice
AST: aspartate transaminase
ALT: alanine transaminase
MS: mercaptosuccinic acid
NAC: N-acetylcystein
AIH: autoimmune hepatitis
GSH: glutathione
GSSG: oxidized glutathione
MDA: malondialdehyde
