# Supplementary figures and images for: Glutathione peroxidase 1 deficiency attenuates concanavalin A-induced hepatic injury by modulation of T-cell activation
Source: Cell Death Dis. 2016 Apr 28;7(4):e2208–. doi: 10.1038/cddis.2016.95 (PMC4855674; doi:10.1038/cddis.2016.95)

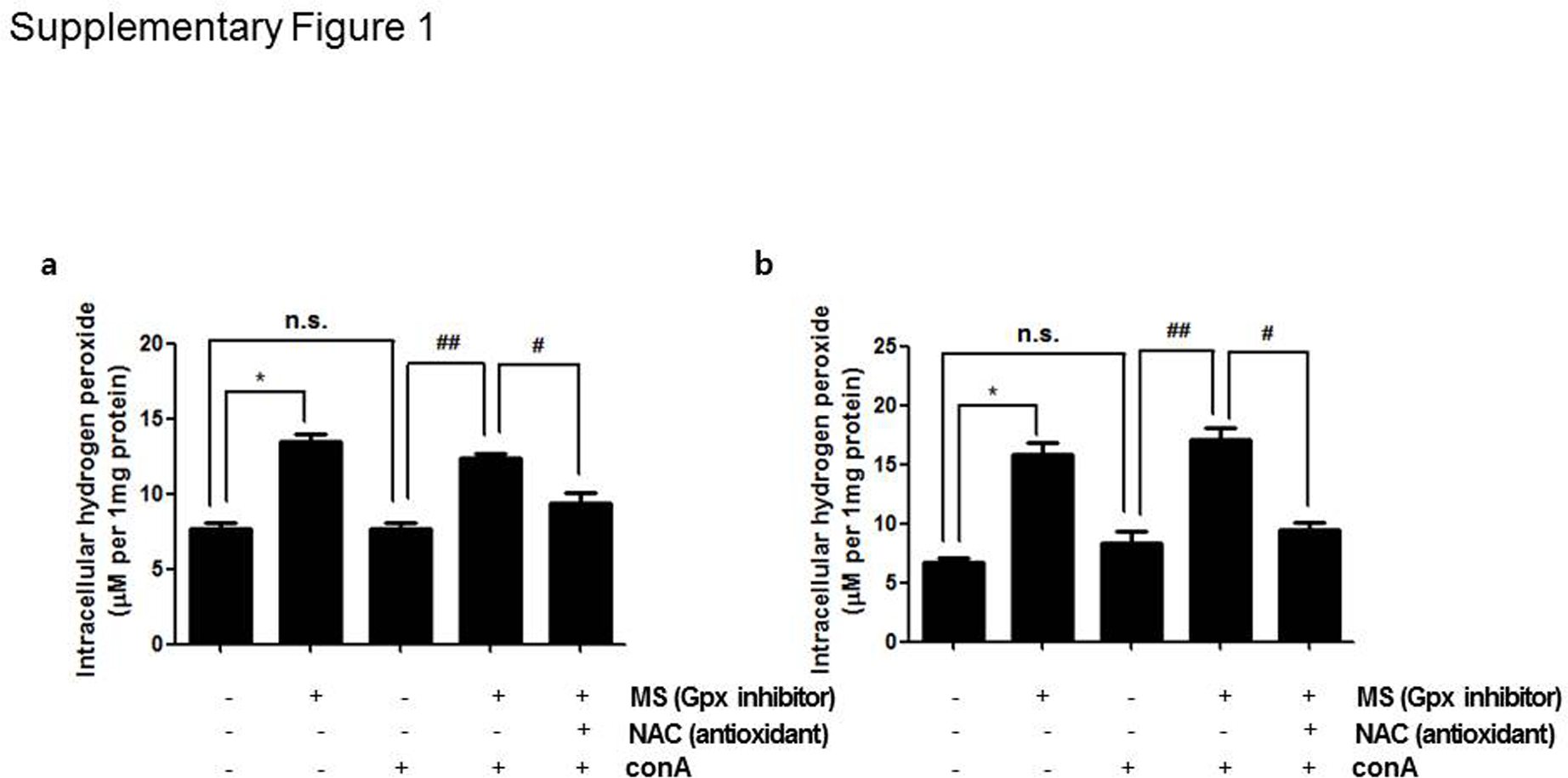

Supplement: Supplementary Figure 1 [file cddis201695x1.tif]
