# Supplementary material for: Glutathione peroxidase 1 deficiency attenuates concanavalin A-induced hepatic injury by modulation of T-cell activation
Source: Cell Death Dis. 2016 Apr 28;7(4):e2208–. doi: 10.1038/cddis.2016.95 (PMC4855674; doi:10.1038/cddis.2016.95)
Supplement: Supplementary Information [file cddis201695x2.doc]

**Supplementary Figure 1. Hydrogen peroxide level was increased by MS but not affected by Con A in spenocytes and Jurkat T cells.** (a) The splenocytes isolated from spleen of WT mice or (b) Jurkat T cells with NAC (5 mM, pharmacological antioxidant) or without, and pretreated MS (0.4 mM) for 48, then treated Con A (5 μg/ ml) for 8h. And we measured hydrogen peroxide level.
